# Predictors of in-hospital mortality in severe leptospirosis: a retrospective cohort study (São Paulo, 2017–2025)

**DOI:** 10.1007/s15010-026-02845-4

**Published:** 2026-06-08

**Authors:** Jorge Júnior Amorim de Freitas, Diego Diovanny Moraes Polonio Nardotto, Lucas Chaves Netto, Shirley Lopes Dias, Yeh-Li Ho

**Affiliations:** 1 Departamento de Infectologia e Doenças Tropicais da Faculdade de Medicina da, Universidade de São Paulo, São Paulo, Brazil; 2 Núcleo de Vigilância Epidemiológica do Hospital das Clínicas da Faculdade de Medicina da, Universidade de São Paulo, São Paulo, Brazil; 3 Divisão de Moléstias Infecciosas e Parasitárias do Hospital das Clínicas da Faculdade de Medicina da, Universidade de São Paulo, São Paulo, Brazil

**Keywords:** Leptospirosis, Weil’s disease, Intensive care, Risk factors, Mortality, Multiorgan failure

## Abstract

**Purpose:**

Although advances in intensive care have improved survival of patients with severe leptospirosis, it remains with high mortality. We aimed to identify factors associated with in-hospital mortality among patients with severe leptospirosis admitted to a tertiary intensive care unit.

**Methods:**

We conducted a retrospective cohort study of adults with leptospirosis admitted to the ICU of a tertiary referral hospital in Brazil, between January 1, 2017, and March 3, 2025. Demographic, clinical, laboratory, and intensive care support variables were collected. The primary outcome was in-hospital mortality. Multivariable logistic regression models were used to identify independent predictors of death.

**Results:**

Forty-four patients were included; median age was 42 years, and 95.5% were male. In-hospital mortality was 20.5% (9/44). Severe multiorgan dysfunction was common; 56.8% of patients required renal replacement therapy and invasive mechanical ventilation in 43.2%. In the clinical multivariable model, alveolar hemorrhage (aOR 7.91, 95% CI 1.07–58.61) and arrhythmia (aOR 13.82, 95% CI 2.06–92.64) were independently associated with death. In the laboratory-based model, urea > 180 mg/dL (aOR 9.01, 95% CI 1.36–59.65) and platelet count < 45 × 10³/mm³ (aOR 14.07, 95% CI 1.40–141.88) were independently associated with mortality.

**Conclusion:**

In this contemporary ICU cohort, mortality in severe leptospirosis was primarily driven by respiratory and cardiac complications rather than isolated renal dysfunction. Although renal failure remained a marker of severity, its prognostic impact appeared attenuated by timely renal replacement therapy.

## Introduction

Leptospirosis is a bacterial zoonosis with global distribution that disproportionately affects populations living under conditions of socioeconomic vulnerability. Caused by pathogenic species of the genus *Leptospira*, the disease is maintained in nature by a wide range of mammalian reservoirs that excrete the bacteria in their urine, perpetuating environmental contamination. Rodents, particularly rats, play a central role in urban transmission cycles. Human infection typically occurs through direct or indirect contact with water or soil contaminated by infected urine, representing a major environmental and occupational health risk worldwide [1, 2].

Estimating the global burden of leptospirosis remains challenging because of underreporting and heterogeneity in diagnostic capacity across regions. A 2015 systematic review and meta-analysis estimated 1.03 million cases and 58,900 deaths annually worldwide, with the greatest burden in tropical and low-income regions. Despite advances in intensive care and supportive management, the World Health Organization (WHO) continues to estimate approximately 60,000 leptospirosis-related deaths each year [1].

In Brazil, leptospirosis is an endemic disease of mandatory notification that may become epidemic during rainy periods, particularly in large urban centers. In 2023, 3,428 cases were confirmed nationwide, with 288 reported deaths [3]. Seasonal outbreaks frequently overlap with dengue epidemics, another major febrile illness, which may delay diagnosis and hinder the timely initiation of appropriate therapy.

Clinically, leptospirosis often presents as a non-specific febrile illness, making early recognition difficult. The disease is classically biphasic, with an initial leptospiemic phase characterized by fever, headache, chills, and myalgia, followed by a transient improvement and an immune-mediated phase that may include aseptic meningitis or uveitis. In approximately 5–10% of symptomatic cases, leptospirosis progresses to a severe multisystem form known as Weil’s disease, which is associated with mortality rates of up to 15%. Hallmark manifestations include acute kidney injury (AKI), jaundice predominantly due to conjugated hyperbilirubinemia, and severe pulmonary involvement, most notably alveolar hemorrhage [4]. Acute respiratory distress syndrome (ARDS) may also occur, sometimes independently of severe hepatic or renal dysfunction, with imaging typically demonstrating bilateral infiltrates consistent with alveolar hemorrhage, ARDS, or pulmonary edema [5].

Pulmonary involvement is the major determinant of prognosis. A prospective observational study reported severe pulmonary disease in approximately 4% of patients with high microscopic agglutination test (MAT) titers, with mortality approaching 70% among those who developed respiratory complications [6]. Coagulation abnormalities further contribute to disease severity, with documented activation of the coagulation cascade, thrombocytopenia, and elevated D-dimer levels, which may progress to disseminated intravascular coagulation and life-threatening hemorrhage [7].

Renal dysfunction is another central feature of severe leptospirosis. A 2024 systematic review and meta-analysis found that approximately 50% of patients develop AKI, nearly 15% require renal replacement therapy, and mortality among those with renal involvement is approximately 12.5% [8]. Importantly, a substantial proportion of survivors subsequently develop chronic kidney disease, highlighting the long-term consequences of severe infection.

Supportive strategies in the intensive care unit (ICU) appear to play a critical role in outcomes. A prospective observational study involving patients with leptospirosis complicated by AKI and ARDS demonstrated significantly lower mortality among those who received early, daily dialysis compared with late, intermittent dialysis [9]. Although limited by its non-randomized design, these findings suggest that timely and intensive organ support may favorably influence outcomes.

Despite these advances, leptospirosis continues to cause substantial morbidity and mortality. Identifying predictors of death and clarifying the role of advanced supportive therapies remain essential to optimize the management of critically ill patients. Accordingly, this study aims to describe the clinical, sociodemographic, and supportive care characteristics of patients with severe leptospirosis admitted to the ICU and to identify risk factors associated with mortality.

## Materials and methods

### Study design

We conducted a retrospective observational study with the aim of identifying factors associated with in-hospital mortality among patients with severe leptospirosis admitted to the intensive care unit (ICU), as well as describing the advanced supportive strategies used in this setting. The study was conducted in a tertiary university hospital (Hospital das Clínicas da Faculdade de Medicina da Universidade de São Paulo—HCFMUSP) in Brazil, a national referral center for the management of severe leptospirosis.

### Participants

Patients admitted between January 1, 2017, and March 3, 2025, with a diagnosis of leptospirosis were assessed. Cases were identified through leptospirosis notification records from the hospital’s Epidemiological Surveillance Unit and were subsequently confirmed by detailed medical record review.

The inclusion criteria were age ≥ 18 years at admission; “highly probable” or “confirmed” cases of leptospirosis – diagnoses were considered confirmed when serological tests (MAT/ELISA) or serum polymerase chain reaction (PCR) were positive; cases were classified as “highly probable” when confirmatory tests were not performed, but clinical and laboratory findings were compatible and no alternative diagnosis was identified; ICU admission for at least 24 h.

Patients were excluded if essential clinical or laboratory data were unavailable or incomplete, precluding adequate analysis.

### Procedures

The data were collected by the authors JJAF and DDMPN. Demographic, clinical, laboratory, and ICU advanced support-related variables were collected. Demographic and severity variables included age, sex, exposure risk, and severity scores (APACHE II and SAPS 3), as well as the presence of comorbidities. Sex was recorded as assigned at birth based on medical records; gender identity data were not available. Exposure risk was classified as high when there was a report of contact with floods or standing water, high-risk occupational activity (such as contact with sewage or waste collection) and/or contact with rodents; low when there was documentation that such contact did not occur; or indeterminate when no description was available.

Clinical variables encompassed baseline and follow-up parameters. At baseline, time intervals from symptom onset to first medical attendance, to first hospital admission, and to admission at HCFMUSP were recorded, as were clinical manifestations. Regarding antimicrobial therapy, the interval between symptom onset and the first dose of antimicrobial and treatment duration were collected. During hospitalization, the occurrence of new organ dysfunction (renal, respiratory, cardiovascular, hepatic, among others) and the timing of its onset were recorded.

At ICU admission, hemodynamic and physiological data were recorded, as well as respiratory parameters, including PaO₂ and the PaO₂/FiO₂ ratio. The need for mechanical ventilation at ICU admission and at any time during hospitalization was also documented. During hospitalization, we recorded the occurrence of oliguria, the need for dialysis, and new-onset arrhythmias, defined as rhythm disturbances first documented during the hospital stay, including atrial fibrillation, paroxysmal supraventricular tachycardia, ventricular tachyarrhythmias, and bradyarrhythmias.

Laboratory data were collected at hospital admission and at ICU admission. These included hemoglobin, hematocrit, leukocytes, and platelets; coagulation profile (INR); urea, creatinine, sodium, and potassium; arterial blood gas analysis (pH and bicarbonate); liver enzymes, total bilirubin and fractions; and creatine phosphokinase (CPK). Respiratory assessment included the need for mechanical ventilation, PaO₂/FiO₂ ratio at ICU admission, the worst PaO₂/FiO₂ ratio observed during hospitalization, and duration of mechanical ventilation. For renal replacement therapy, when indicated, parameters immediately before dialysis initiation were recorded (including urea, creatinine, sodium, potassium, calcium, and urine output), as well as cumulative fluid balance in the first 3 ICU days. Additionally, the maximum values of AST, ALT, direct bilirubin, indirect bilirubin, and lipase during hospitalization were extracted.

The ICU advanced supportive strategies assessed included hemodynamic support (use of vasoactive drugs), ventilatory support (PaO₂/FiO₂ ratio in admission, the worst PaO₂/FiO₂ ratio, need for mechanical ventilation and duration of mechanical ventilation), renal replacement therapy (renal replacement therapy modality and renal parameters/urine output before dialysis), and use of blood products (packed red blood cells and platelets). Alveolar hemorrhage was defined by the presence of diffuse bilateral pulmonary infiltrates associated with reported hemoptysis and/or aspiration of bloody secretions through the endotracheal tube.

### Outcome

The primary outcome was hospital mortality. The secondary outcome was to determine the risk factors for mortality and comparison between the patients who received dialysis versus those who did not.

### Statistical analysis

Data were initially entered into a Microsoft Excel spreadsheet and subsequently analyzed using IBM SPSS Statistics (version 21). A 5% significance level (*p* < 0.05) was adopted, with estimates presented with 95% confidence intervals (95% CI). Normality of continuous variables was assessed using the Shapiro–Wilk test. Data with a normal distribution were expressed as mean ± SD, while non-normally distributed data were expressed as median and interquartile range (IQR). Categorical variables were expressed as counts and percentages. Binary clinical variables and interventions, including oliguria, dyspnea, cough, hemoptysis, arrhythmia, need for mechanical ventilation, and dialysis, were also evaluated.

In univariable analyses, the association between categorical variables and mortality was assessed using the chi-square test or the Fisher exact test, as appropriate and crude odds ratios (ORs) and corresponding 95% CIs were estimated. Quantitative variables were compared using Student’s t test for normal distributions or the Mann–Whitney test for non-normal distributions.

Some continuous variables were subsequently categorized for association analyses with the outcome, based on clinical cutoffs or according to the best accuracy point on the ROC curve, defined by the Youden index for prediction of death: APACHE II (> 17), platelets (< 45,000/mm³), urea (> 180 mg/dL), creatinine (> 5.0 mg/dL), bicarbonate (< 20 mEq/L). The presence of oliguria, dyspnea, cough, hemoptysis, arrhythmia, and the need for mechanical ventilation (at admission and during hospitalization) and dialysis, among others, were also analyzed as dichotomous variables.

Given the limited number of events, a parsimonious approach was adopted for multivariable model building to reduce the risk of overfitting. Variables with *p* < 0.05 in univariable analysis and/or considered clinically relevant were selected as candidates for logistic regression. A forward stepwise approach was used to assess the stability of the model (including standard errors), the width of confidence intervals, and the presence of near-perfect separation between predictors and outcome. Results were presented as adjusted odds ratios (aORs), with 95% CIs and corresponding p values.

### Role of the funding source

This study received no external funding. No funding source had any role in the study design; data collection, analysis, or interpretation; writing of the report; or the decision to submit the manuscript for publication.

## Results

A total of 44 patients were included. Median age was 42 years (IQR 26.8–58.3), and 95.5% were male, most of whom had no previously known comorbidities. Nearly all patients were classified as having a high exposure risk (79.5%). At ICU admission, disease severity was moderate to high, with a mean APACHE II score of 17.3 (SD 7.77) and a mean SAPS 3 score of 65.18 (SD 15.89) (Table 1).

The median time from symptom onset to first medical attendance was three days (IQR 1–6), five days (IQR 4–6.8) to first hospital admission, and 6.5 days (IQR 5–8) to admission to the tertiary hospital. The most frequent presenting symptoms were jaundice (100%), myalgia (90.9%), fever (81.8%), dark urine (70.5%), calf pain (65.9%), and oliguria (61.4%), whereas respiratory symptoms were less common (Table 1).

At hospital admission, laboratory tests showed anemia and marked thrombocytopenia (median hemoglobin 10.55 g/dL [IQR 10.0–12.3]; median platelet count 44,500/mm³ [28,250–76,750]). Renal dysfunction was prominent (mean urea 165.9 mg/dL [SD 66.0] and mean creatinine 4.94 mg/dL [SD 2.21]), accompanied by metabolic acidosis (median pH 7.36 [IQR 7.31–7.40]). Biochemical evidence of cholestasis was common, with marked hyperbilirubinemia (total bilirubin 13.98 mg/dL [IQR 8.36–22.72]) (Table 1).

**Table 1:** Demographic, clinical and laboratory characteristics of patients at admission

	Total (44)	Survivors (35)	Death (9)
Demographic characteristics			
Age, years	42 (26.75–58.25)	40 (26–52)	56 (30–59)
Sex (male)	95.5%	100%	94.30%
Exposure risk	High: 35 (79.5%); Low: 1 (2.3%); Indeterminate: 8 (18.2%)	High: 30 (85.7%); Low: 1 (2.9%); Indeterminate: 4 (11.4%)	High: 5 (55.6%); Low: 0 (0%); Indeterminate: 4 (44.4%)
APACHE II	17.3 ± 7.77	15.57 ± 6.93	24 ± 7.53
SAPS 3	65.18 ± 15.89	62.31 ± 15.19	76.33 ± 14.19
Comorbidities			
Diabetes mellitus	0 (0%)	0 (0%)	0 (0%)
Hypertension	4 (9.1%)	4 (11.4%)	0 (0%)
Cardiovascular disease	1 (2.3%)	1 (2.9%)	0 (0%)
Chronic kidney disease	0 (0%)	0 (0%)	0 (0%)
Chronic liver disease	1 (2.3%)	0 (0%)	1 (11.1%)
AIDS	2 (4.5%)	1 (2.9%)	1 (11.1%)
Chronic lung disease	1 (2.3%)	1 (2.9%)	0 (0%)
Symptoms			
Time from symptom onset to first medical contact, days	3 (1–6)	3 (1–5)	6 (3–11.5)
Time from symptom onset to first hospital admission, days	5 (4–6.75)	5 (4–6)	6 (5.5–11.5)
Time from symptom onset to admission at HCFMUSP, days	6.5 (5–8)	6 (5–8)	7 (5.5–12)
Fever	36 (81.8%)	28 (80%)	8 (88.9%)
Myalgia	40 (90.9%)	33 (94.3%)	7 (77.8%)
Calf pain	29 (65.9%)	24 (68.6%)	5 (55.6%)
Headache	20 (45.5%)	17 (48.6%)	3 (33.3%)
Oliguria	27 (61.4%)	19 (54.3%)	8 (88.9%)
Jaundice	44 (100%)	35 (100%)	9 (100%)
Cough	12 (27.3%)	7 (20%)	5 (55.6%)
Hemoptysis	12 (27.3%)	8 (22.9%)	4 (44.4%)
Dyspnea	14 (31.8%)	7 (20%)	7 (77.8%)
Abdominal pain	23 (52.3%)	18 (51.4%)	5 (55.6%)
Nausea or vomiting	26 (59.1%)	22 (62.9%)	4 (44.4%)
Laboratory tests at hospital admission			
Hemoglobin, g/dL	10.55 (10.0–12.3)	10.7 (10.2–12.3)	10.3 (8.2–11.95)
Hematocrit, %	29.80 ± 6.06	30.33 ± 5.75	27.73 ± 7.11
Leukocyte count, /mm³	15 245 (11 410–19 155)	15 150 (11 370–19 200)	16 240 (10 735–20 855)
Platelet count, /mm³	44 500 (28 250–76 750)	54 000 (33 000–77 000)	29 000 (18 500–41 000)
INR	1.1 (1.0–1.16)	1.1 (1.0–1.16)	1.03 (0.99–1.29)
Urea, mg/dL	165.91 ± 65.97	156.26 ± 66.39	203.44 ± 51.67
Creatinine, mg/dL	4.94 ± 2.21	4.49 ± 2.18	6.67 ± 1.34
Sodium, mEq/L	136.98 ± 5.41	137.5 ± 5.14	135.11 ± 6.29
Potassium, mEq/L	3.9 ± 0.92	3.84 ± 0.89	4.45 ± 0.94
Arterial pH	7.355 (7.31–7.40)	7.360 (7.324–7.420)	7.287 (7.024–7.364)
Bicarbonate, mEq/L	19.74 ± 4.88	20.7 ± 4.69	15.8 ± 3.68
AST, U/L	125 (79–170)	104.5 (73.75–152.75)	170 (92.5–439.5)
ALT, U/L	80 (61–134)	78 (62.5–125.5)	100 (51.0–204.5)
Total bilirubin, mg/dL	13.98 (8.36–22.72)	13.88 (7.14–22.57)	14.08 (9.13–23.01)
Direct bilirubin, mg/dL	11.90 (6.36–19.42)	11.48 (5.9–19.21)	12.4 (8.47–21.78)
Indirect bilirubin, mg/dL	1.36 (0.74–3.47)	1.32 (0.72–3.8)	1.60 (0.58–1.68)
Creatine kinase, U/L	901 (293.5–2642.5)	726 (256.5–1599.5)	2190 (985.75–17 480.75)

Data are presented for the overall cohort and stratified by survival status (survivors vs. death). Continuous variables are expressed as mean ± SD or median (IQR), according to data distribution, and categorical variables as n (%). Exposure risk was classified as high, low, or indeterminate based on epidemiological history. Laboratory values correspond to measurements obtained at hospital admission. APACHE II=Acute Physiology and Chronic Health Evaluation II. SAPS 3 = Simplified Acute Physiology Score 3. AIDS=acquired immunodeficiency syndrome. INR=international normalized ratio. AST=aspartate aminotransferase. ALT=alanine aminotransferase. HCFMUSP=Hospital das Clínicas da Faculdade de Medicina da Universidade de São Paulo

Antimicrobial therapy was initiated at a median of 5.5 days (IQR 4–7) after symptom onset and was maintained for a median of seven days (IQR 7–8). Hemorrhagic complications were frequent, including alveolar hemorrhage (43.2%), bleeding at puncture sites (22.7%), and upper gastrointestinal bleeding (11.4%), with high transfusion requirements for packed red blood cells (43.2%) and platelets (29.5%). Invasive mechanical ventilation was required in 43.2% of patients and vasopressors in 52.3%. Mean PaO₂/FiO₂ at ICU admission was 325.8 (SD 157.69), and the mean worst PaO₂/FiO₂ during hospitalization was 168.7 (SD 73.9). Renal replacement therapy was required in 56.8% of patients, most commonly on a daily schedule (38.6%), with a median time to initiation of one day of hospital admission (IQR 0–1) and a median duration of five days (IQR 3–14). Cumulative fluid balance over the first three ICU days was − 46 mL (IQR − 2021 to 1666). Cardiac arrhythmia occurred in 25% of patients. Overall in-hospital mortality was 20.5% (Table 2).

**Table 2:** Clinical complications, intensive care interventions, and outcomes

	Total (44)	Survivors (35)	Death (9)
Treatment			
Time from symptom onset to antimicrobial therapy, days	5.5 (4–7)	5 (4–7)	7 (5.5–11)
Duration of antimicrobial therapy, days	7 (7–8)	7 (7–8)	5 (2–7)
Hematological complications			
Upper gastrointestinal bleeding	5 (11.4%)	2 (5.7%)	3 (33.3%)
Alveolar hemorrhage	19 (43.2%)	12 (34.3%)	7 (77.8%)
Bleeding at puncture site	10 (22.7%)	6 (17.1%)	4 (44.4%)
Packed red blood cell transfusion	19 (43.2%)	12 (34.3%)	7 (77.8%)
Platelet transfusion	13 (29.5%)	8 (22.9%)	5 (55.6%)
Respiratory complications			
Need for mechanical ventilation	19 (43.2%)	10 (28.6%)	9 (100%)
PaO₂/FiO₂ ratio at ICU admission	325.75 ± 157.69	345.68 ± 138.31	254.56 ± 210.70
Worst PaO₂/FiO₂ ratio	168.71 ± 73.94	161.5 ± 57.97	166.5 ± 96.15
Renal complications			
Need for renal replacement therapy	25 (56.8%)	18 (51.4%)	7 (77.8%)
Pre-dialysis laboratory tests			
Urea, mg/dL	204.35 ± 63.98	193.94 ± 66.44	228.14 ± 55.0
Creatinine, mg/dL	6.71 (4.92–7.58)	6.46 (4.87–7.32)	7.07 (6.27–7.64)
Bicarbonate, mEq/L	18.04 ± 3.00	18.46 ± 2.63	17.07 ± 3.76
Sodium, mEq/L	138.52 ± 6.01	138.19 ± 6.63	139.28 ± 4.64
Potassium, mEq/L	4.33 ± 0.89	4.13 ± 0.78	4.77 ± 1.03
Ionized calcium, mg/dL	4.36 ± 0.44	4.43 ± 0.46	4.20 ± 0.36
Urine output before dialysis, mL/h	15 (1.40–66.65)	17.5 (2.1–71.22)	12.5 (0–18.75)
Time to dialysis initiation, days	1 (0–1)	0.5 (0–1)	1 (0–1)
Duration of dialysis, days	5 (3–14)	5 (3–14)	5 (4–16)
Cumulative fluid balance during first 3 ICU days, mL	-46 (-2021 – +1666)	-399 (-2243 – +1497)	+ 1542 (-405 – +7421)
Cardiac complications			
Arrhythmia	11 (25%)	5 (14.3%)	6 (66.7%)
Need for vasoactive drugs	23 (52.3%)	14 (40%)	9 (100%)
Other laboratory abnormalities			
Peak AST, U/L	136 (87–206.5)	123 (76–157)	292 (149.5–18773)
Peak ALT, U/L	116.5 (78.5–167.75)	119 (78–167)	114 (72–4045)
Peak direct bilirubin, mg/dL	19.00 ± 10.11	18.59 ± 9.68	20.59 ± 12.10
Peak indirect bilirubin, mg/dL	2.19 (1.03–4.49)	1.85 (0.99–4.63)	2.8 (1.32–4.91)
Peak lipase, U/L	193.5 (87–421.25)	167.0 (81.5–367.75)	449.5 (125.25–922.0)
Outcome			
In-hospital mortality	9 (20.5%)		

Data are shown for the overall cohort and stratified by survival status (survivors vs. death). Continuous variables are presented as mean ± SD or median (IQR), according to data distribution, and categorical variables as n (%). Antimicrobial treatment variables include time from symptom onset to initiation of therapy and total duration of antimicrobial use. Respiratory, renal, hematological, and cardiac complications, as well as intensive care interventions, were recorded during the ICU stay. Pre-dialysis laboratory parameters and urine output correspond to measurements obtained immediately before initiation of renal replacement therapy. Cumulative fluid balance refers to the net balance during the first three days of ICU admission. Outcomes are reported as in-hospital mortality. ICU=intensive care unit. PaO₂/FiO₂=ratio of arterial oxygen partial pressure to fraction of inspired oxygen. AST=aspartate aminotransferase. ALT=alanine aminotransferase

Patients requiring renal replacement therapy differed from those who did not in baseline characteristics, severity, organ dysfunction, and ICU support (Table 3). They were significantly older (median age 52 years [IQR 37.5–59.5] vs. 32 years [IQR 24–48]; *p* = 0.018) and exhibited greater severity at admission, with higher APACHE II (20.24 vs. 13.42; mean difference 6.88, 95% CI 2.48 to 11.16; *p* = 0.003) and SAPS 3 scores (71.12 vs. 57.37; mean difference 13.75, 95% CI 4.85 to 22.65; *p* = 0.003). Additionally, oliguria was more frequent (80.0% vs. 36.8%; *p* = 0.005), and they had more severe renal and metabolic abnormalities at admission, including higher urea (188.4 vs. 136.3 mg/dL; *p* = 0.008), higher creatinine (5.9 vs. 3.68 mg/dL; *p* < 0.001), and higher potassium (4.29 vs. 3.53 mmol/L; *p* = 0.006), and greater severity of metabolic acidosis (Table 3).

Hemorrhagic complications were more frequent among patients who required dialysis, including alveolar hemorrhage (60.0% vs. 21.1%; *p* = 0.014) and bleeding at puncture sites (36.0% vs. 5.3%; *p* = 0.027), requiring more blood component transfusions. These patients also more frequently required invasive mechanical ventilation (64.0% vs. 15.8%; *p* = 0.002) and vasopressors (76.0% vs. 21.1%; *p* = 0.001). During hospitalization, they reached higher peaks of cholestasis and lipase (Table 3). In-hospital mortality was higher among patients who required dialysis, although this difference did not reach statistical significance (28.0% vs. 10.5%; *p* = 0.26) (Table 3).

**Table 3:** Comparison of demographic, clinical, laboratory, and outcome characteristics between patients who received renal replacement therapy and those who did not

	No dialysis (19)	With dialysis (25)	*p*
Age, years	32 (24–48)	52 (37.5–59.5)	0.018
Sex (male)	18 (94.7%)	24 (96%)	1
APACHE II	13.42 ± 6.6	20.24 ± 7.3	0.003
SAPS 3	57.37 ± 14.8	71.12 ± 14.2	0.003
Symptoms			
Time from symptom onset to first medical contact, days	2 (1–6)	4 (1.5–6)	0.382
Time from symptom onset to first hospital admission, days	5 (4–7)	5 (4–6)	0.9
Time from symptom onset to admission at HCFMUSP, days	6 (5–8)	7 (5–8.5)	0.911
Fever	17 (89.5%)	19 (76.0%)	0.433
Myalgia	17 (89.5%)	23 (92%)	1
Calf pain	12 (63.2%)	17 (68.0%)	0.759
Headache	8 (42.1%)	12 (48%)	0.766
Oliguria	7 (36.8%)	20 (80%)	0.005
Dark urine	15 (78.9%)	16 (64%)	0.335
Jaundice	19 (100%)	25 (100%)	-
Hemoptysis	4 (21.1%)	8 (32%)	0.507
Dyspnea	3 (15.8%)	11 (44%)	0.058
Abdominal pain	9 (47.4%)	14 (56%)	0.761
Nausea or vomiting	11 (57.9%)	15 (60%)	1
Laboratory tests at hospital admission			
Hemoglobin, g/dL	11.1 (10.0–12.4)	10.5 (9.9–12.2)	0.569
Hematocrit, %	30.64 ± 6.36	29.16 ± 5.87	0.428
Leukocyte count, /mm³	15 800 (11 040–21 040)	15 150 (12 185–19 110)	0.743
Platelet count, /mm³	53 000 (33 000–134 000)	42 000 (24 000–64 500)	0.165
INR	1.05 (0.9700–1.1600)	1.11 (1.0025–1.1675)	0.59
Urea, mg/dL	136.32 ± 51.49	188.4 ± 67.75	0.008
Creatinine, mg/dL	3.68 ± 2.02	5.9 ± 1.86	< 0.001
Sodium, mEq/L	136 ± 5.27	137.7 ± 5.49	0.301
Potassium, mEq/L	3.53 ± 0.86	4.29 ± 0.84	0.006
Arterial pH	7.400 (7.370–7.435)	7.3245 (7.2585–7.3588)	< 0.001
Bicarbonate, mEq/L	22.17 ± 5.96	18.02 ± 3.05	0.006
AST, U/L	92 (64–159)	143.5 (99.25–192.5)	0.093
ALT, U/L	89 (47–136)	78 (63.75–130.75)	0.966
Total bilirubin, mg/dL	11.56 (6.70–21.43)	14.80 (10.285–23.67)	0.29
Direct bilirubin, mg/dL	10.65 (5.89–18.43)	12.92 (7.585–21.285)	0.384
Indirect bilirubin, mg/dL	0.93 (0.53–2.22)	1.60 (0.85–3.725)	0.298
Creatine kinase, U/L	574.5 (278.75–992.75)	1246 (354–2892)	0.113
Treatment			
Time from symptom onset to antimicrobial therapy, days	6 (4–8)	5 (4–7)	0.939
Duration of antimicrobial therapy, days	7 (7–8)	7 (6.5–8)	0.848
Hematological complications			
Upper gastrointestinal bleeding	1 (5.3%)	4 (16%)	0.37
Alveolar hemorrhage	4 (21.1%)	15 (60%)	0.014
Bleeding at puncture site	1 (5.3%)	9 (36%)	0.027
Packed red blood cell transfusion	3 (15.8%)	16 (64%)	0.002
Platelet transfusion	2 (10.5%)	11 (44%)	0.021
Respiratory complications			
Need for mechanical ventilation	3 (15.8%)	16 (64%)	0.002
PaO₂/FiO₂ ratio at ICU admission	357 ± 129	306 ± 173	0.381
Worst PaO₂/FiO₂ ratio	174 ± 44	162 ± 79	0.844
Renal parameters			
Cumulative fluid balance during first 3 ICU days, mL	-1072 (-2664 – +370)	+ 259 (-1779 – +2001)	0.120
Cardiac complications			
Arrhythmia	3 (15.8%)	8 (32%)	0.301
Need for vasoactive drugs	4 (21.1%)	19 (76%)	0.001
Other laboratory abnormalities			
Peak AST, U/L	93 (71–159)	147 (104.5–245)	0.041
Peak ALT, U/L	119 (66–187)	114 (83.5–157)	0.995
Peak direct bilirubin, mg/dL	15.43 ± 10.2	21.71 ± 9.3	0.039
Peak indirect bilirubin, mg/dL	1.17 (0.63–2.93)	2.8 (1.635–5.365)	0.01
Peak lipase, U/L	92.5 (54.75–165.5)	353.5 (219.75–470.5)	< 0.001
Outcomes			
In-hospital mortality	2 (10.5%)	7 (28%)	0.26

Data are presented according to the use of renal replacement therapy (no dialysis vs. with dialysis). Continuous variables are expressed as mean ± SD or median (IQR), according to data distribution, and categorical variables as n (%). P values refer to univariable comparisons between groups. Laboratory parameters correspond to measurements obtained at hospital admission, unless otherwise specified. Peak laboratory values represent the highest value recorded during hospitalization. Cumulative fluid balance refers to the net balance during the first three days of ICU admission. Outcomes are reported as in-hospital mortality. APACHE II=Acute Physiology and Chronic Health Evaluation II. SAPS 3 = Simplified Acute Physiology Score 3. INR=international normalized ratio. AST=aspartate aminotransferase. ALT=alanine aminotransferase. PaO₂/FiO₂=ratio of arterial oxygen partial pressure to fraction of inspired oxygen. ICU=intensive care unit. HCFMUSP=Hospital das Clínicas da Faculdade de Medicina da Universidade de São Paulo

In univariable analysis, mortality was associated with greater disease severity (APACHE II > 17; OR 12.0; 95% CI 1.35–106.81; *p* = 0.021) and significant associations were observed for arrhythmia (OR 18.0; 95% CI 2.80–115.56; *p* = 0.002), severe thrombocytopenia (platelets < 45,000/mm³; OR 10.67; 95% CI 1.20–94.74; *p* = 0.023), urea > 180 mg/dL (OR 7.64; 95% CI 1.36–42.90; *p* = 0.021), creatinine > 5.0 mg/dL (OR 13.54; 95% CI 1.52–120.85; *p* = 0.008), and bicarbonate < 20 mmol/L (OR 8.40; 95% CI 0.93–76.15; *p* = 0.050) (Table 4).

**Table 4:** Univariable associations between categorical clinical variables and in-hospital mortality

	*N*	Deaths *n* (%)	Odds ratio (95% CI)	*p*-value
APACHE II > 17				
Yes	22	8 (36.4)	12.00 (1.35–106.81)	0.02
No	22	1 (4.5)	Reference	
Oliguria				
Yes	27	8 (29.6)	6.73 (0.76–59.75)	0.12
No	17	1 (5.9)	Reference	
Alveolar hemorrhage				
Yes	19	7 (36.8)	6.71 (1.20–37.40)	0.027
No	25	2 (8.0)	Reference	
Dyspnea				
Yes	14	7 (50.0)	14.00 (2.37–82.71)	0.02
No	30	2 (6.7)	Reference	
Hemoptysis				
Yes	12	4 (33.3)	2.70 (0.58–12.51)	0.23
No	32	5 (15.6)	Reference	
Mean arterial pressure < 65 mmHg				
Yes	6	3 (50.0)	5.33 (0.86–32.99)	0.09
No	38	6 (15.8)	Reference	
Mechanical ventilation				
Yes	19	9 (47.4)	Not estimable*	< 0.001
No	25	0 (0.0)	Reference	
Arrhythmia				
Yes	11	6 (54.5)	18.00 (2.80–115.56)	0.002
No	32	2 (6.3)	Reference	
Renal replacement therapy				
Yes	25	7 (28.0)	3.31 (0.60–18.19)	0.26
No	19	2 (10.5)	Reference	
Platelet count < 45 × 10³/mm³				
Yes	22	8 (36.4)	12.0 (1.35–106.80)	0.021
No	22	1 (4.5)	Reference	
Bicarbonate < 20 mEq/L				
Yes	22	7 (31.8)	8.40 (0.93–76.15)	0.05
No	19	1 (5.3)	Reference	
Urea > 180 mg/dL				
Yes	18	7 (38.9)	7.64 (1.36–42.90)	0.021
No	26	2 (7.7)	Reference	
Creatinine > 5.0 mg/dL				
Yes	21	8 (38.1)	13.54 (1.52–120.85)	0.008
No	23	1 (4.3)	Reference	
Cumulative fluid balance at 72 h > 1000 ml				
Yes	12	5 (41.7)	6.78 (1.06–43.36)	0.071
No	21	2 (9.5)	Reference	

Data are presented as n (%) unless otherwise specified. Odds ratios (ORs) with 95% CIs were calculated using univariable logistic regression to assess the association between selected categorical variables and in-hospital mortality. For each variable, the absence of the characteristic was used as the reference category. Laboratory and clinical variables refer to values obtained at hospital or ICU admission, unless otherwise specified. Cumulative fluid balance corresponds to the net balance at 72 h of ICU admission. APACHE II=Acute Physiology and Chronic Health Evaluation II. ICU=intensive care unit.* Odds ratio not estimable because of complete separation due to absence of deaths in the reference group

To minimize overfitting and avoid collinearity among variables reflecting similar organ dysfunctions, two separate multivariable models were developed: one comprising clinical variables and the other laboratory variables. In the clinical model (model 1), alveolar hemorrhage (adjusted OR 7.91, 95% CI 1.07–58.61; *p* = 0.043) and arrhythmia (adjusted OR 13.82, 95% CI 2.06–92.64; *p* = 0.007) remained independently associated with death (Table 5). In the laboratory-based model (model 2), three variables reflecting renal–metabolic dysfunction reached statistical significance in univariable analysis (*p* < 0.05). Urea was prioritized for inclusion in the multivariable model given the recognized contribution of uremia to bleeding risk and its broader systemic impact. In this model, urea > 180 mg/dL (adjusted OR 9.01, 95% CI 1.36–59.65; *p* = 0.023) and platelets < 45 000/mm³ (adjusted OR 14.07, 95% CI 1.40–141.88; *p* = 0.025) were independently associated with death (Table 5).

**Table 5:** Multivariable logistic regression analysis of clinical and laboratory variables associated with in-hospital mortality

Model 1 (clinical variables)	aOR (95% CI)	*p*
Alveolar hemorrhage	7.914 (1.069–58.611)	0.043
Arrhythmia	13.817 (2.061–92.643)	0.007

Model 2 (laboratory variables)		
Urea > 180 mg/dL	9.012 (1.361–59.652)	0.023
Platelet count < 45 000/mm³	14.068 (1.395–141.884)	0.025

Adjusted odds ratios (aORs) with 95% CIs were obtained using multivariable logistic regression to identify independent clinical and laboratory predictors of in-hospital mortality. The models included clinically and laboratory relevant variables selected a priori based on biological plausibility and results from univariable analyses. Associations are reported as aORs with corresponding p values, aOR=adjusted odds ratio; CI=confidence interval

## Discussion

This cohort was composed predominantly of young adult men with high exposure risk, consistent with Brazilian and international reports of severe leptospirosis. This marked male predominance likely reflects greater occupational, environmental, and social vulnerability-related exposure to contaminated water, mud, soil, sewage, and waste, particularly during floods and heavy rainfall events. In our setting, these exposures commonly affect men working in waste collection, agriculture, and other outdoor or manual occupations, as well as people experiencing homelessness or living in vulnerable urban areas.

The low prevalence of chronic comorbidities suggests that disease severity is driven primarily by the intensity of the infectious insult and the host inflammatory response rather than by underlying chronic disease [10]. The high frequency of classical clinical features of Weil’s disease combined with severe laboratory abnormalities indicates delayed presentation, with patients already exhibiting advanced disease at the time of admission to tertiary care. This is supported by the substantial interval between symptom onset, first medical contact, and referral hospital admission, which likely contributed to greater severity at ICU admission.

Despite the inclusion of exclusively ICU-admitted patients with high severity scores, the observed mortality rate of 20.5% was relatively low. Mortality rates reported in the literature vary widely across settings and periods, suggesting that prognosis reflects the interaction between baseline severity and timely access to advanced supportive care. Earlier hospital-based studies reported case fatality rates of 15–18% [11, 12]. In contrast, Delmas et al. demonstrated that a protocol advocating immediate ICU transfer after initial evaluation was associated with a marked reduction in ICU mortality to 6%, reinforcing the importance of early intervention and ICU availability in modulating outcomes [13].

Renal and metabolic abnormalities were prominent at ICU admission. Median urea and creatinine levels were consistent with AKIN stage 3 acute kidney injury, and bicarbonate levels indicated severe metabolic acidosis. Nevertheless, 43% of patients did not require renal replacement therapy (RRT). Even among those managed without dialysis, renal dysfunction remained significant, with median urea and creatinine levels of 138 mg/dL and 3.68 mg/dL, respectively, corresponding to at least AKIN stage 2, along with marked thrombocytopenia. These findings raise important questions regarding the timing and indications for dialysis in Weil’s disease.

Historically, acute kidney injury—particularly oliguria—has been a major prognostic determinant in severe leptospirosis, and hyperkalemia has also been associated with mortality [11, 12]. In contemporary ICU cohorts, however, the prognostic impact of renal failure may be attenuated by timely access to RRT. In the cohort reported by Delmas et al., neither creatinine levels nor the need for RRT was associated with mortality, suggesting that adequate renal support may mitigate the lethal consequences of AKI [13]. Nonetheless, the concept of “early dialysis” in Weil’s disease remains poorly defined.

In the clinical trial by Andrade et al., intensive daily dialysis was associated with lower mortality compared with a conservative strategy. However, all included patients had advanced renal dysfunction and concomitant respiratory failure requiring mechanical ventilation, underscoring the coexistence of multiorgan dysfunction [9]. Similarly, in our cohort, the indication for dialysis was influenced not only by renal and metabolic parameters but also by dysfunction in other organ systems, including oliguria, alveolar hemorrhage, and the need for vasoactive support, resulting in particularly high severity scores. Notably, RRT use was not associated with mortality in univariable analysis, supporting the notion that dialysis decisions should be guided by overall clinical context, multiorgan involvement, and volume status rather than isolated laboratory thresholds. Once indicated, RRT should be initiated promptly, with modality and intensity tailored to the degree of renal dysfunction, metabolic derangement, and fluid overload [9, 14].

Respiratory involvement emerged as a key determinant of outcome. The finding of alveolar hemorrhage as an independent risk factor for death reinforces the prognostic relevance of respiratory failure in severe leptospirosis. Unlike renal dysfunction, respiratory failure remains particularly challenging to manage, especially in resource-limited settings. Previous studies have consistently linked pulmonary manifestations—including dyspnea, crackles, and alveolar infiltrates—to worse prognosis [11, 12, 15, 16]. In the cohort of Delmas et al., refractory ARDS was present in most fatal cases, suggesting that progression to severe pulmonary failure may be associated with a narrow window of reversibility [13].

The use of extracorporeal membrane oxygenation (ECMO) has increasingly been reported in patients with ARDS secondary to Weil’s disease. A recent narrative review encompassing 43 cases reported an overall survival rate of 84%, indicating that ECMO is feasible and potentially beneficial in selected patients [17]. In our series, all three patients who required ECMO survived.

Leptospirosis has also been investigated as a potential contributor to chronic kidney disease, particularly in endemic regions such as Latin America, where recurrent or severe infection may contribute to the burden of renal disease [8]. Although nearly half of the patients in our cohort required renal replacement therapy during hospitalization, none of the survivors remained dialysis-dependent after discharge. However, because long-term follow-up and post-discharge serum creatinine measurements were not systematically available, we were unable to determine the incidence of chronic kidney disease in this cohort. Future prospective studies should assess long-term renal outcomes after severe leptospirosis, particularly among patients requiring renal replacement therapy during the acute phase.

We observed an association between arrhythmia and mortality, which may reflect myocardial involvement as well as the effects of electrolyte disturbances and acidosis. Previous studies have identified myocarditis and arrhythmias as independent risk factors for severity and death in Weil’s disease [18–20].

This study has limitations, including its retrospective design, limited sample size, and small number of deaths, which reduce statistical power and limit multivariable adjustment. These constraints preclude causal inference and increase the risk of type II error. In addition, the use of corticosteroids or other immunosuppressive agents as adjunctive therapy aimed at modulating the host immune response was not systematically evaluated; therefore, we could not assess their efficacy or association with clinical outcomes.

Future studies should better define optimal timing and intensity of RRT using detailed pre-dialysis data, and systematically evaluate ventilation strategies in patients with severe pulmonary involvement, ideally in prospective cohorts adjusted for disease severity.

In summary, our findings suggest a shift in the prognostic hierarchy in the ICU era. Renal dysfunction remains a key marker of severity, particularly when accompanied by oliguria, but its impact may be mitigated by timely supportive care and RRT. In contrast, severe respiratory failure has emerged as the principal driver of mortality in critically ill patients with leptospirosis, underscoring the need for early recognition and aggressive management of pulmonary complications.

## Data Availability

Deidentified individual participant data and a data dictionary will be available upon reasonable request to the corresponding author from the time of publication, subject to approval of a proposal and a data use agreement. The study protocol and statistical analysis plan will be available upon request.

## Declarations

### Artificial intelligence

During the preparation of this manuscript, generative artificial intelligence tools were used to assist with grammar, clarity, and language editing. The authors critically reviewed and edited the content and take full responsibility for the accuracy, integrity, and final version of the manuscript.
